# Short-Term High-Fat Diet Fuels Colitis Progression in Mice Associated With Changes in Blood Metabolome and Intestinal Gene Expression

**DOI:** 10.3389/fnut.2022.899829

**Published:** 2022-06-07

**Authors:** Zhen-Hua Wu, Jing Yang, Lei Chen, Chuang Du, Qi Zhang, Shan-Shan Zhao, Xiao-Yu Wang, Jing Yang, Yang Liu, Demin Cai, Jian Du, Hui-Xin Liu

**Affiliations:** ^1^Health Sciences Institute, China Medical University, Shenyang, China; ^2^Institute of Life Sciences, China Medical University, Shenyang, China; ^3^Liaoning Key Laboratory of Obesity and Glucose/Lipid Associated Metabolic Diseases, China Medical University, Shenyang, China; ^4^Department of Endocrinology, The Fourth Affiliated Hospital, China Medical University, Shenyang, China; ^5^Laboratory of Animal Physiology and Molecular Nutrition, College of Animal Science and Technology, Yangzhou University, Yangzhou, China

**Keywords:** nutrition, metabolism, homeostasis, imaging mass microscope, inflammatory bowel disease

## Abstract

Clinical cases and animal experiments show that high-fat (HF) diet is involved in inflammatory bowel disease (IBD), but the specific mechanism is not fully clear. A close association between long-term HF-induced obesity and IBD has been well-documented. However, there has been limited evaluation of the impact of short-term HF feeding on the risk of intestinal inflammation, particularly on the risk of disrupted metabolic homeostasis. In this study, we analyzed the metabolic profile and tested the vulnerability of 2,4,6-trinitrobenzenesulfonic acid (TNBS)-induced colitis after short-term HF feeding in mice. The results showed that compared with the control diet (CD), the fatty acid (FA), amino acid (AA), and bile acid (BA) metabolisms of mice in the HF group were significantly changed. HF-fed mice showed an increase in the content of saturated and unsaturated FAs and a decrease in the content of tryptophan (Trp). Furthermore, the disturbed spatial distribution of taurocholic acid (TCA) in the ileum and colon was identified in the HF group using matrix-assisted laser desorption/ionization-mass spectrometry imaging (MALDI-MSI). After HF priming, mice on TNBS induction were subjected to more severe colonic ulceration and histological damage compared with their CD counterparts. In addition, TNBS enema induced higher gene expressions of mucosal pro-inflammatory cytokines under HF priming conditions. Overall, our results show that HF may promote colitis by disturbing lipid, AA, and BA metabolic homeostasis and inflammatory gene expressions.

## Introduction

It is well established that the diet and the microbiome can contribute to the occurrence of metabolic diseases in part by causing intestinal inflammation and increased permeability (1). Our previous studies and others show that a long-term high-fat (HF) diet causes nutritional imbalance, resulting in obesity, insulin resistance, and other diseases (2–4). HF intake can cause the disorder of lipid metabolism and induce systemic chronic low-grade inflammation, and the colon may be the first organ affected by inflammation caused by HF (5, 6). The intestinal mucosa is the largest interface between the body itself and the external environment, which has barrier functions such as selective infiltration and absorption of nutrients and defense against the invasion of microorganisms and inflammatory factors in the intestine (7). The altered intestinal environment could influence metabolic homeostasis, especially the metabolism of amino acids (AAs), fatty acids (FAs), and bile acids (BAs) (8–10).

Although the exact etiology of inflammatory bowel disease (IBD) is not fully understood, nutrition and dietary factors, in particular HF, have been recognized to play an important role in the pathogenesis of IBD (11). IBD comprises Crohn’s disease and ulcerative colitis, which are characterized by chronic and relapsing inflammation of the gastrointestinal tract (12). IBD has become a global disease with accelerating incidence in newly industrialized countries whose societies have become more westernized, and this increase has paralleled a “westernization” of lifestyle (13). Many studies have described the relationship between fat intake and IBD pathogeny. Several studies have investigated the development of colitis in long-term HF-fed animals (14, 15). Studies have shown that long-term HF consumption will destroy the intestinal immune homeostasis and induce inflammation in animal models, and epidemiological studies have also shown that excessive HF intake is closely related to the occurrence and relapse of IBD (16, 17). A high intake of unsaturated fats may be associated with an increased risk of ulcerative colitis (18). However, most of the previous studies have ignored the effect of HF on the serum metabolites and intestines before disease induction. Furthermore, the effects of short-term HF feeding in colitis and the underlying molecular mechanisms at the levels of metabolism profile need to be further explored. In this context, a better understanding of the pathogenesis of short-term HF-driven metabolic disorders may help to reduce the IBD burden worldwide.

In this study, we proposed a 4-week HF priming to evaluate the effects of short-term fat intake on the risk of inflammatory diseases. Metabolomic and gene expression investigation results indicate that BA, FA, and AA metabolisms are significantly reprogrammed in the HF-fed group. In addition, mass spectrometry microscopy discovered that HF-feeding disturbs the spatial distribution of BAs and causes the decrease of taurocholic acid (TCA) in the intestinal wall, which may weaken the ability of the intestinal mucosal barrier to resist the invasion of bacteria, toxins, and antigens under inflammatory state. Moreover, under short-term HF priming conditions, 2,4,6-trinitrobenzenesulfonic acid (TNBS) administration aggravates the severity of colitis companied with dysregulated metabolism.

## Materials and Methods

### Animals and Experimental Treatment

All animal procedures were approved by the Institutional Animal Care and Use Committee (IACUC) at the China Medical University. Notably, 6–8 weeks old male C57BL/6 mice (*n* = 10 per group) were purchased from Beijing HFK Biotechnology Co., Ltd. All experimental mice were housed in specific pathogen-free environments under a controlled condition of normal circadian circulation for 12 h at 20–22°C and 45 ± 5% humidity, with free access to food and water. Mice were fed an HF diet (TP23520, Trophic Diet, China) or a control diet (CD, TP23524, Trophic Diet, China) for 4 weeks. HF contained 60% available energy as fat, 20% available energy as carbohydrate, and 20% available energy as protein. CD contained 10% available energy as fat, 70% available energy as carbohydrate, and 20% available energy as protein. After 4 weeks of feeding, colitis was induced using the reported TNBS (P2297-10 ml, sigma)-colitis model with some modifications (19). In brief, mice were fed with HF or CD for 3 weeks and then pre-sensitized with 150 μl of 1% (wt/vol) TNBS solution applied to the back skin for 8 days. Next, the animals were fasted overnight and treated under anesthesia with a 100 mg/kg mixture of 5% TNBS and 100% alcohol (1:1) *via* intrarectal injection, and the control mice received 50% alcohol treatment. Then, the mice were placed upside down for 5 min after TNBS injection. Body weight, stool consistence, and rectal bleeding were monitored daily. Animals were sacrificed on the third day after TNBS treatment. During the experimental period, the food intake of mice was recorded two times a week, and the body weights were documented per week.

### Sample Collection

Animals were sacrificed, and the blood was collected with anticoagulant and then centrifuged at 1,000 × *g* for 10 min at 22–25°C for serum collection. Then, the liver, ileum, colon, and colon content were carefully dissected and kept in liquid nitrogen before storage at −80°C. Besides, parts of the colons and livers were harvested for histological analysis.

### Biochemical Analysis

The analysis of triglyceride (TG), total cholesterol (TC), low-density lipoprotein cholesterol (LDL-c), high-density lipoprotein cholesterol (HDL-c), aspartate aminotransferase (AST), alanine aminotransferase (ALT), and total bile acid (TBA) were quantified using commercial kits (Nanjing Jiancheng Bioengineering Institute, Nanjing, China) in accordance with manufacturer’s instructions.

### Colon Histology Assay

After the mice were euthanized, the colons were quickly removed and rolled up using the “swiss roll” method (20). Then, the colons were fixed in 4% neutral formalin for 24 h and embedded in paraffin. The embedded tissue blocks were cut into 4 μm sections and stained with hematoxylin and eosin (H&E). The histological injury was evaluated according to the existing standard (16, 21).

### RNA Extraction and qPCR

Total RNAs were extracted using TRIzol reagent (Invitrogen), and then the extracted total RNAs were reverse-transcribed into cDNA using the PrimeScript RT reagent kit (TaKaRa, Mountain View, CA, United States) according to the manufacturer’s instructions. The relative expression levels of genes were calculated using the 2^–ΔΔCT^ formula (22), and GAPDH was chosen as an internal control. The primers are listed in Supplementary Table 1.

### Metabolomics

Bile acids, FAs, and AAs were quantified as previously described methods (23, 24). In brief, AAs and FAs were quantified by HPLC coupled to tandem mass spectrometry (MS/MS) based on deuterated purified standards. Serum AA and FA concentrations were expressed in μmol/L and mmol/L, respectively.

### Sample Preparation for Imaging Mass Microscope

Frozen 10 μm of mouse intestinal sections were sliced at −20°C with a cryomicrotome (Leica CM1950, Nussloch, Germany) and then thaw-mounted onto electrically conductive glass slides. Subsequently, a “two-step matrix application,” which combined with sublimation and airbrushing, was used to coat the matrix (9AA) for tissue sections.

### Imaging Mass Spectrum Analysis Based on Imaging Mass Microscope

iMScope was performed using a 1,000 Hz solid laser. A 40-μm pitch of special resolution was used, and the data were acquired in negative ionization. The *m*/*z* values were internally calibrated with DHB. All the spectra were acquired using atmospheric pressure matrix-assisted laser desorption/ionization (MALDI) (Shimadzu Corporation). The laser in the iMScope system was a diode-pumped 355 nm Nd: YAG laser (Shimadzu Corporation, Kyoto, Japan) and operated under the following parameters: frequency, 1,000 Hz; laser intensity, 55.0; laser diameter, 3 μm. The parameters of IT-TOF MS were set as follows: ion polarity, negative; mass range, 250–550; sample voltage, 3.0 kV; detector voltage, 1.90 kV. The imaging MS Solution Version 1.30 software (Shimadzu, Tokyo, Japan) was used to control the instrument, and the data acquisition, visualization, and quantification were also performed using the same software.

### Tissue Preparation for Histology After Mass Spectrometry Imaging

The tissue sections were stained with H&E for examination following the previous protocol (25). In brief, the matrix (9AA) covered on the glass slides was removed with 70% ethanol, and then the tissues were fixed with 100% ethanol. The tissues were then stained with H&E. Finally, the H&E slides were sealed with neutral gum and scanned using an iMScope TRIO (Shimadzu, Japan) instrument.

### Statistical Analysis

Data in bar graphs are expressed as mean ± SEM. The unpaired two-tailed Student’s *t*-test and the two-tailed Wilcox test were used to compare two groups of independent samples. The Kruskal–Wallis ANOVA test was utilized to determine significance in multiple groups. The SPSS Statistics version 25.0 software and the GraphPad Prism 8 software were used for statistical analyses. All statistical tests with a *p*-value of <0.05 were considered statistically significant.

## Results

### Lipid Metabolism-Related Parameters in Serum, Liver, Ileum, and Colon of Mice Fed With High-Fat Diet

**FIGURE 1:**
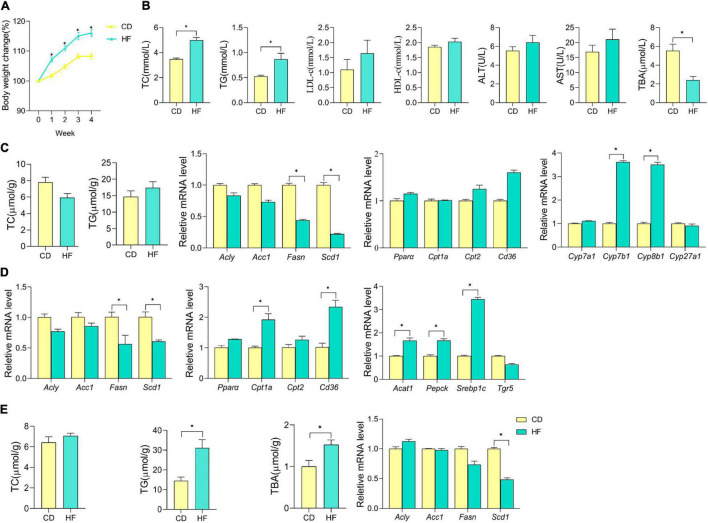
Effect of HF on the blood, liver, ileum, and colon lipid metabolism in mice. **(A)** Body weight changes in mice on CD or HF for 4 weeks. **(B)** Cholesterol (TC), triglyceride (TG), low-density lipoprotein cholesterol (LDL-c), high-density lipoprotein cholesterol (HDL-c), alanine aminotransferase (ALT), and aspartate aminotransferase (AST) contents in the serum of each group of mice. **(C)** Cholesterol (TC) and triglyceride (TG) contents in the liver of each group of mice; hepatic mRNA expression of genes involved in lipid metabolism and BA synthesis, and data were from seven pooled samples of each group. **(D)** Ileum mRNA expression of genes involved in lipid and carbohydrate metabolism in each group of mice (data were from seven pooled samples of each group). **(E)** Cholesterol (TC), triglyceride (TG), and total bile acid (TBA) contents in the colon; colonic mRNA expression of genes involved in lipid metabolism in each group of mice (data were from seven pooled samples of each group). *n* ≥ 6. The data were shown as mean ± SEM. *p < 0.05. CD, control diet group; HF, high-fat diet group.

We have noticed that the impact of HF on the weight of mice was detectable within the first week of dietary intake. Compared with the CD, the HF statistically increased the body weight of mice at the end of this dietary treatment (*p* < 0.05) (Figure 1A). Abnormal levels of lipid metabolism-related parameters in the serum, liver, ileum, and colon were observed among mice fed with HF. As shown in Figure 1B, HF-fed mice had an increase in serum TC and TG levels and a decrease in serum TBA levels than CD-fed mice (*p* < 0.05), and no significant differences were observed in the serum levels of LDL-c, HDL-c, ALT, and AST between CD and HF groups. Meanwhile, there was no significant difference in the TC and TG levels between CD and HF group livers (Figure 1C). To understand the effect of HF on metabolism at the genetic level, we examined the changes in several related genes in CD and HF groups by qPCR. The expression levels of genes associated with hepatic FA synthesis (*Fasn*, *Scd1*) were downregulated in the HF group compared with those in the CD group (*p* < 0.05) (Figure 1C), but the expression of key genes involved in BA synthesis (*Cyp7b1*, *Cyp8b1*) was upregulated in HF fed livers (*p* < 0.05). Moreover, 4 weeks of HF feeding might disturb carbohydrate and lipid metabolism in the mouse ileum, which was indicated by the changes of key genes involved in the above-mentioned pathways at mRNA levels (Figure 1D). Interestingly, HF feeding prominently increased the contents of TG in colonic mucosa and the content of TBA in colonic feces compared with CD feeding (*p* < 0.05) (Figure 1E). In the colon, the expression levels of *Scd1* were downregulated in the HF group compared with those in the CD group (*p* < 0.05) (Figure 1E). Taken together, these results highly suggested that HF feeding rendered the disturbance of metabolic homeostasis.

### Dysbiosis of Serum Metabolic Patterns in High-Fat Diet Feeding Mice

**FIGURE 2:**
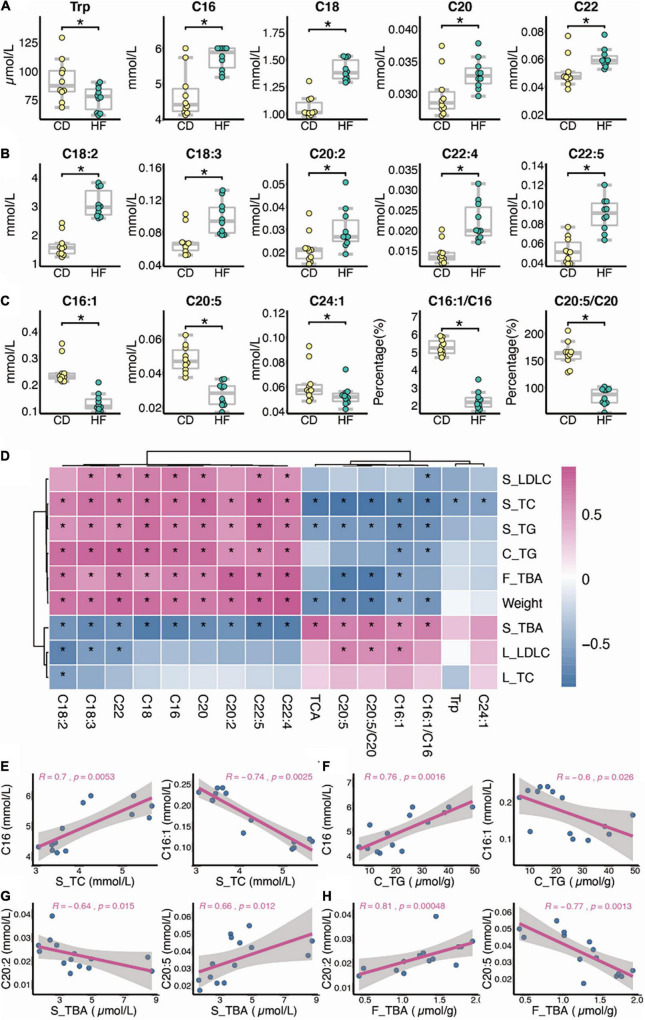
Dysbiosis of serum metabolic patterns in HF feeding mice. **(A–C)** Differential metabolites between CD and HF groups (*n* = 10). **(D–H)** Spearman correlation between changed metabolites and biochemical indexes (*n* = 7; S, serum; L, liver; C, colon; F, feces). The two-tailed Wilcox test was used to determine the significant difference in CD and HF groups. *p < 0.05. CD, control diet group; HF, high-fat diet group.

As shown in Figures 2A–C, regarding the effect of HF feeding on serum FA and AA levels analyzed at week 4, the levels of nine FA species were higher (*p* < 0.05), namely, C16, C18, C20, C22, C18:2, C18:3, C20:2, C22:4, and C22:5, while the levels of three FAs and one AA species were lower (*p* < 0.05), namely, C16:1, eicosapentaenoic acid (EPA, namely, C20:5), C24:1, and tryptophan (Trp), in HF as compared with CD mice. In addition, the serum *Scd1* desaturation index (C16:1/C16) was decreased significantly in the HF-fed mice (*p* < 0.05), and we further noticed that the percentage of C20:5/C20 also decreased in HF feeding mice (*p* < 0.05). Through Spearman correlation between changed metabolites and biochemical indexes, as shown in Figures 2D–H, overall serum LDL-c, TC, TG levels, colon TG level, feces TBA level, and body weight were positively correlated with nine FA species, namely, C18:2, C18:3, C22, C18, C16, C20, C20:2, C22:4, and C22:5, besides they were negatively correlated with TCA, C20:5, C20:5/C20, C16:1, C16:1/C16, Trp, and C24:1. Moreover, for serum TBA level, positive correlation with TCA, C20:5, C20:5/C20, C16:1, and C16:1/C16 and a negative correlation with C18:2, C18:3, C22, C18, C16, C20, C20:2, C22:4, and C22:5 were observed.

### High-Fat Diet Decreases the Spatial Distribution of Taurocholic Acid in the Ileum and Colon of Mice

**FIGURE 3:**
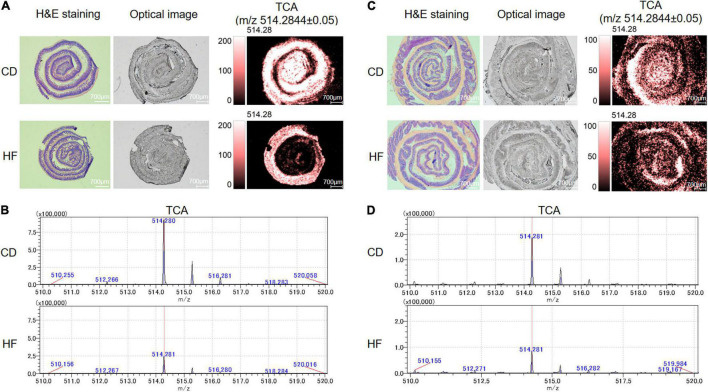
Detection of TCA distributed within the regions of the ileum and colon by MALDI-MSI. **(A)** MS ion images of the spatial distribution of TCA from ileum tissue. Experimental *m*/*z* values were presented in parentheses. TCA (*m*/*z* 514.2844 ± 0.05). All ion images were normalized to the 9-AA matrix signal. **(B)** MALDI-MSI single-pixel mass spectra of TCA from ileum tissue. **(C)** MS ion images of the spatial distribution of TCA from colon tissue. Experimental *m*/*z* values were presented in parentheses. TCA (*m*/*z* 514.2844 ± 0.05). All ion images were normalized to the 9-AA matrix signal. **(D)** MALDI-MSI single-pixel mass spectra of TCA from colon tissue. CD, control diet group; HF, high-fat diet group.

Recently, we developed a method for identification and spatial visualization of dysregulated BA metabolism in HF-fed mice by mass spectrometry imaging (MSI) (26). The spatially resolved profiling of the altered BA metabolism was detected in the HF group with the most significant changes in TCA. In this study, we focused on the detection of TCA due to its biological activity on anti-inflammation (26, 27). As the MSI technique putative identification is only based on the measured exact *m*/*z* value, we used secondary mass spectrometry to distinguish isomers of target compounds with the same molecular formula by MSI (e.g., *m*/*z* 514.2844 ± 0.05, putative identification as TCA/TMCA). The mass spectra of the TCA standard are shown in Supplementary Figure 1A, with a negative ion scan at *m*/*z* 514.284 as a [M-H]^–^ peak. Secondary mass spectrometry analysis of the *m*/*z* 514.284 ion yielded *m*/*z* 353.247 and *m*/*z* 496.272 fragment ions. These two fragment ion peaks can be regarded as the characteristic ion peaks of TCA. We performed secondary mass spectrometry analysis on ileum and colon tissue sections (shown in Supplementary Figures 1B,C) and also found characteristic fragment ion peaks at *m*/*z* 353.247 and *m*/*z* 496.272. Therefore, we confirmed that the material on the ileum and colon was TCA. BAs were ionized in negative mode, and all ion images were normalized to the 9AA matrix signal. Thus, we cut down a 3-cm long terminal ileum and whole colon and rolled them up in a “swiss roll” (20). The spatial distribution of TCA in ileum and colon sections is shown in Figures 3A,C. The ion intensity of TCA in ileum and colon tissue sections of HF-treated mice dramatically decreased when compared with the CD group’s MS ion image. Figures 3B,D show the mass spectra of TCA in the ileum and colon, respectively. These data indicated that HF-treated mice suffered a more severe decrease of TCA in colonic tissue, and we speculated that this is associated with HF exacerbating colitis.

### Changes of Metabolic Patterns in Control Diet and High-Fat Diet Feeding Mice After 2,4,6-Trinitrobenzenesulfonic Acid Installation

**FIGURE 4:**
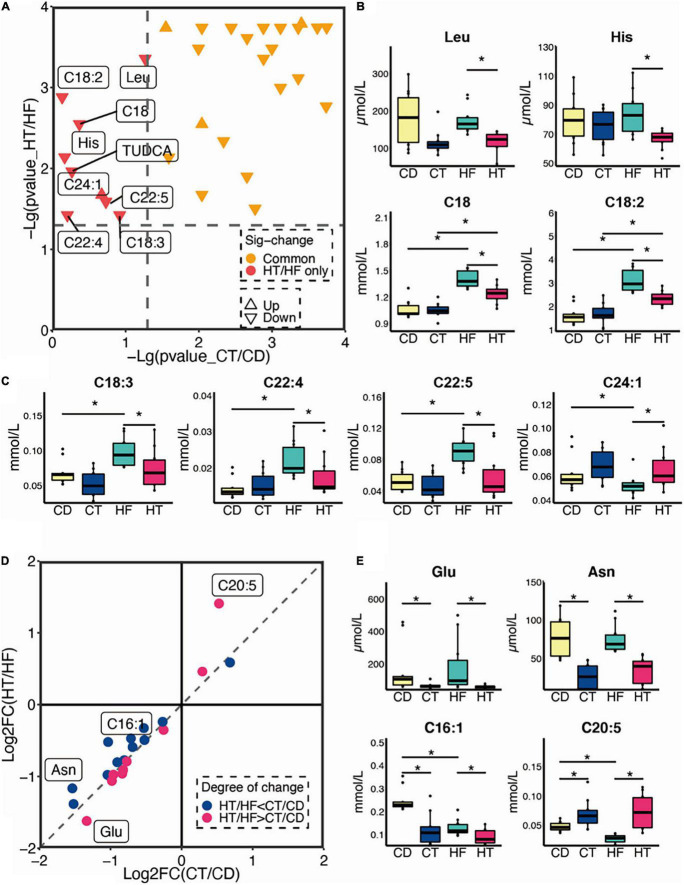
Changes in metabolic patterns in CD and HF feeding mice after being treated with TNBS. **(A)** Distribution of significantly differential serum metabolites in comparisons of CT and CD and HT and HF (*n* = 10). **(B,C)** The unique change of metabolites between the HT and HF groups rather than in the CT and CD groups. **(D,E)** The degree of change in common metabolites when compared with CT and CD and HT and HF. The Kruskal–Wallis ANOVA test was utilized to determine the significant difference in multiple groups. *p < 0.05. CD, control diet group; HF, high-fat diet group; CT, control diet and TNBS group; HT, high-fat diet and TNBS group.

Following TNBS installation, we found that the levels of alanine (Ala), asparagine (Asn), glycine (Gly), isoleucine (Ile), leucine (Leu), lysine (Lys), methionine (Met), serine (Ser), threonine (Thr), tryptophan (Trp), valine (Val), tyrosine (Tyr), glutamine (Gln), and proline (Pro) in serum were significantly lower in the two TNBS groups than their counterparts (data not shown). Notably, the distribution of significantly differential serum metabolites in the respective comparisons of CT and CD (the CT group represents CD-fed mice, which are treated with TNBS) and HT and HF (the HT group represents HF-fed mice, which are treated with TNBS) is shown in Figure 4A. The metabolites represented by the red triangle in the upper left corner are specific to the comparison between HT and HF, including Leu, histidine (His), C18, C18:2, C18:3, C22:4, C22:5, C24:1, and TUDCA, and these metabolites were significantly changed in the HF group instead of the CD group after TNBS enema (Figure 4A). Additionally, the metabolites represented by the orange triangle in the upper right corner were both changed when compared with CT and CD and HT and HF, which were not identified in detail (Figure 4A). Furthermore, the unique change of metabolites between the HT and HF groups rather than the CD and CT groups is shown in Figures 4B,C. The data showed that C24:1 was upregulated in the HF group after TNBS treatment (*p* < 0.05), but the other metabolites were downregulated (*p* < 0.05) (Figures 4B,C). As for the commonly changed metabolites when we compared CT and CD and HT and HF, we have especially noticed that glutamic acid (Glu) and C20:5 showed a greater degree of change in HT and HF than in CT and CD, but the Asn and C16:1 showed a greater degree of change in CT and CD than HT and HF (Figure 4D). In addition, Glu, Asn, and C16:1 were decreased following TNBS installation, and C20:5 was increased (Figure 4E).

### Alternation of Serum Metabolism Between CT and HT Groups

**FIGURE 5:**
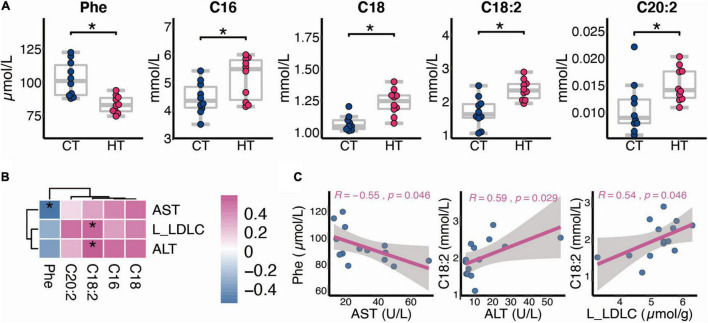
Alternation of serum metabolism between the CT and HT groups. **(A)** Differential metabolites between the CT and HT groups (*n* = 10). **(B,C)** Spearman correlation between changed metabolites and biochemical indexes (*n* = 7, L, liver). The two-tailed Wilcox test was used to determine the significant difference in CT and HT groups. *p < 0.05. CT, control diet and TNBS group; HT, high-fat diet and TNBS group.

Data from our targeted metabolomics suggested that HF affects the AAs and FAs after TNBS treatment. As shown in Figure 5A, compared with the CT group, phenylalanine (Phe) decreased in the HT group (*p* < 0.05), whereas C16, C18, C18:2, and C20:2 increased in the HT group (*p* < 0.05). Besides, liver LDL-c level and serum ALT activity were positively correlated with C18:2, and serum AST activity was negatively correlated with Phe (Figures 5B,C).

### High-Fat Diet Aggravated the Disease Severity in 2,4,6-Trinitrobenzenesulfonic Acid-Induced Colitis

**FIGURE 6:**
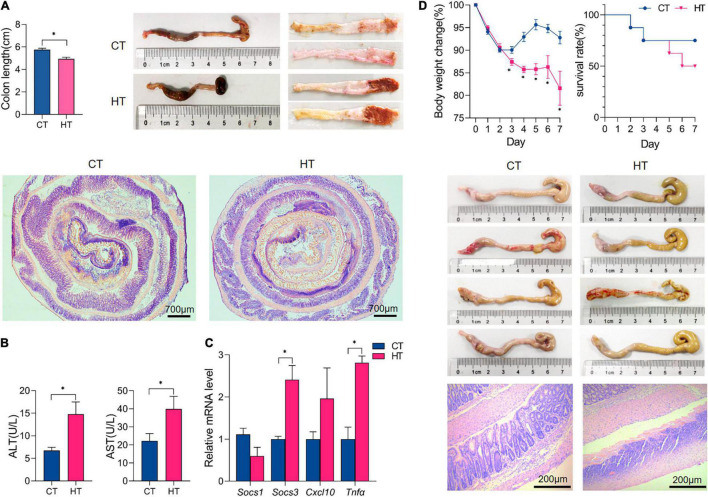
High-fat exacerbated the disease severity of TNBS-induced colitis. **(A)** All mice were sacrificed on day 3, and the colons were collected to estimate mucosal damage by detecting colon lengths; gross morphology of the colons on day 3 after TNBS treatment; the representative histological sections were observed under microscopy (magnification: 2.5×). **(B)** The alanine aminotransferase (ALT) and aspartate aminotransferase (AST) activity in the serum of each group of mice. **(C)** qPCR quantitation of pro-inflammatory cytokines and chemokines in colonic mucosa on day 3 after TNBS treatment. **(D)** Body weight changes for each group of mice within 7 days after TNBS installation and survival curve within 7 days; gross morphology of the colons on day 7 after TNBS treatment; and colon histology by hematoxylin and eosin staining on day 7 (magnification: 100×). *n* ≥ 3. The data were shown as mean ± SEM. *p < 0.05. CT, control diet and TNBS group; HT, high-fat diet and TNBS group.

As shown in Supplementary Figure 2A, male C57BL/6 mice were primed with an HF, and CD primed mice were used as normal control. Three weeks later, mice of the model group were pre-sensitized with 1% (wt/vol) TNBS solution, and 8 days later, mice were treated with 2.5% (wt/vol) TNBS solution *via* intrarectal injection for 3 days to induce colitis. Several studies have shown that mice fed an HF diet showed increased levels of inflammatory cytokines (*Tnf*α and *Il6*) in the ileum, colon, and surrounding mesenteric fat, even before the development of obesity (28, 29). In this study, we found that *Cxcl10* was elevated in the colonic tissue of mice fed an HF for 4 weeks (*p* < 0.05), and *Tnf*α, *Socs1*, and *Socs3* were not changed (Supplementary Figure 2D). It is common knowledge that *Cxcl10* mainly induces the chemotaxis of monocytes and macrophages, participates in regulating the migration, activation, and differentiation of a variety of immune cells, and affects acquired immunity and inflammation response (30). Therefore, the colon tissue in the HF group may have low-grade inflammation, which may be a reason for the more serious colitis after TNBS treatment compared with the CD group. Following TNBS installation, HF-primed mice caused much more severe colitis than mice of CD, as evidenced by a significant decrease in the body weight and shortening of colon length (*p* < 0.05) and significantly higher colonic ulceration and histological damage (Figures 6A,D). Then, we measured AST and ALT activities in serum. As shown in Figure 6B, we have noticed that the HT group had higher AST and ALT activities than the CT group (*p* < 0.05); moreover, no obvious histological alterations in H&E staining were observed in the liver of the HT group when compared with the CT group (data not shown). It is known that ALT and AST can sensitively reflect whether hepatocytes are damaged or not and the degree of injury. Importantly, as shown in Figure 6C, HF-treated mice expressed much higher levels of pro-inflammatory cytokines and chemokines (including *Tnf*α and *Cxcl10*) in the colonic mucosa compared with CD-treated mice following TNBS induction (*p* < 0.05). Cytokines, such as *Tnf*α, are known to play key roles in the induction of gut inflammation and tumorigenesis (31). In addition, we further studied the effect of HF on TNBS-induced colitis within 7 days. As shown in Figure 6D, CD-primed mice developed weight loss following TNBS treatment, and the loss reached the maximum on days 2–3 followed by a gradual recovery, but it decreased again on days 5 until day 7. In contrast, the weight loss of HF-primed mice was more severe and was almost not recovered within 7 days (*p* < 0.05) (Figure 6D), approximately 50% of HF-primed mice died within 7 days after TNBS treatment (Figure 6D), and during this period, the mortality of CD-primed mice was only 25% after TNBS treatment (Figure 6D). Following TNBS installation, the colons from the surviving HF-primed mice remained short by day 7 compared with CD-primed mice after TNBS treatment (Figure 6D), and the colons were swollen with no visible fecal pellet formation in both groups. Moreover, histological examination revealed severe ulceration in the colon of the HT group (Figure 6D). These data indicated that HF-primed mice suffered more severe colonic inflammation than CD-primed mice. Taken together, HF-primed mice showed dysregulated metabolic homeostasis, which might promote the disease severity in TNBS-induced colitis.

## Discussion

The incidence of metabolic-related diseases has gradually increased throughout the world, such as diabetes, hypertension, and IBD, especially in emerging market countries with gradually westernized eating habits, which is thought closely related to the increase in HF intake (6, 32). Previous studies show that due to the imbalance between energy intake and expenditure, long-term HF could cause severe disorders of metabolism, which highly increased susceptibility to the development of metabolic diseases (33, 34). Meanwhile, accumulating evidence shows that the involvement of HF increases in intestinal levels of secondary BAs, characterized by the substantial increase of DCA in the feces, might be highly relevant to the pathogenesis of IBD (35, 36). HF consumption promotes and exacerbates experimental colitis in dietary and genetic mouse models of IBD (37). Taken together, dietary fats play an important role in intestinal disease pathogenesis. However, most of these studies examined the relationship between westernized diets and the pathogenesis of metabolic-related diseases and focused on secondary effects of HF, such as effects on gut microbes or BAs (38, 39). The current study focuses on the specific effects of short-term HF feeding on the levels of free FAs, AAs, and BAs in serum and the resulting effects on colitis development. In this study, we discovered that 1 month of HF feeding disturbed FA and AA metabolisms in serum and caused the reduction of the spatial distribution of TCA in the ileum and colon wall of mice, which may result in increasing the lipid peroxidation and the weakening of the resistance of colon wall to pathogens such as bacteria, thus aggravating epithelial barrier dysfunction and colonic mucosal inflammation. Consistently, we found that short-term HF-priming deteriorated TNBS-induced colitis, proved by more violent mucosal inflammation and broader colonic damage compared with CD-primed mice after TNBS treatment. These data indicated that short-term HF priming distributed metabolism, which led to increased susceptibility and severity of IBD.

For only 4 weeks of HF-feeding in mice, not only the blood lipid (TC and TG) increased but also TG accumulated in the colon, which may lead to mitochondrial dysfunction, oxidative stress, and other damage. In addition, the content of total BAs in mice colonic feces increased significantly, which is similar to previous reports that HF increased the production of secondary BAs (40). Compared with other internal organs, the liver is considered to be prone to fat accumulation (41), but our results showed that short-term (4 weeks) HF feeding did not cause significant fat accumulation in the liver. An interesting feature of gene expression was the suppression of enzymes involved in lipid synthesis, and these included *Fasn* and *Scd1*. It is worth noting that similar phenomena were observed in the ileum and colon. *Scd1* is considered one of the key enzymes in lipid homeostasis and body weight regulation (42). Our studies have shown that HF feeding decreased mRNA level and desaturation index of *Scd1*. The decrease of *Scd1* may cause lipid acylation disorder and change the lipid composition of the cell membrane, resulting in serious lipid toxicity (43).

Metabolomic investigations show that Trp was significantly lower in the serum of mice on HF than in the control group. Trp is one of the important essential AAs (EAAs). Research shows that disorders in Trp metabolism results in lower levels of bacterial-derived and beneficial metabolites, and some Trp metabolites can provide protection against gastroenteric effects and IBD (1, 44). For instance, Trp exerts a beneficial regulatory function in mucosal growth or maintenance and alleviation of intestinal inflammation by the 5-hydroxytryptophan (5-HT) signaling pathway (45). Other studies also suggest that Trp plays a role in the recovery of colitis and in the function of intestinal homeostasis by caspase recruitment domain family member 9 (Card9), calcium-sensing receptor (CaSR), and aryl hydrocarbon receptor (AHR) ligands in the intestine (46–48). Therefore, the significant decrease of serum Try in mice fed an HF for 1 month may be a risk factor for TNBS-induced colitis. Furthermore, saturated FAs are non-essential FAs, and excessive intake will increase the content of blood lipids in the body. Our results show that the increase of C16, C18, C20, and C22 in serum is closely related to dyslipidemia caused by HF. It should be noted that compared with the CD group, essential FAs C18:2, C18:3, C20:2, C22:4, and C22:5 were significantly increased in the HF group. Although we have known that polyunsaturated FAs positively affect insulin sensitivity, cardiovascular, mental health, and development and reduce hypertension and inflammation (49), the increase of serum-free FAs will lead to systemic low-grade inflammation, and the increase of HF-derived free FAs in the intestinal cavity will lead to the increased production of pro-inflammatory cytokines in the intestinal tract (50). These results indicated that HF intake had a regulating effect on FA metabolisms. Thus, we speculated that polyunsaturated FAs are a double-edged sword, and their advantages and disadvantages depend on the specific physiological conditions and reasonable physiological concentration of the body. Further study on their particular physiological significance is needed in the short-term HF feeding model. Notably, palmitoleic acid (C16:1) and EPA decreased in the HF group. Lipogenesis is mediated by *Scd1*, the rate-limiting enzyme catalyzing the synthesis of monounsaturated FAs, and the predominant substrates for *Scd1* are palmitic (C16) and stearic acids (C18) which generate C16:1 and oleic acid (C18:1), respectively (49). The reduction of C16:1 and increase of C16 and C18 are consistent with the decreased expression of *Scd1* in the liver in our results. C16:1 is a monounsaturated FA and has therapeutic effects on some chronic diseases such as metabolic syndrome, diabetes, and inflammation (49). EPA is an important polyunsaturated FA, also known as arachidonic acid and deep-sea fish oil, which belongs to the ω-3 series of polyunsaturated FAs. It is an important and indispensable nutrient that cannot be synthesized by the human body itself. EPA is known to have a variety of health benefits including well-established hypotriglyceridemic, antioxidant, and anti-inflammatory effects (49). It could be of interest to human health and the prevention of cardiovascular disease (51). Therefore, the decrease of C16:1 and EPA in the HF group may be conducive to the production of metabolic diseases.

Following TNBS installation, the distribution of significantly differential serum metabolites also changed. We did not find any metabolites changed significantly which only occurred in the CD and its colitis model. Importantly, the serum levels of Leu, His, C18, C18:2, C18:3, C22:4, and C22:5 are decreased significantly when we compared HT with HF instead of CT and CD, which may be the result of the joint action of HF and TNBS. Studies have shown that branched-chain AAs (e.g., Leu, Val, and Ile) supplementation with protein-restricted diet improved intestinal immune defense function by protecting villous morphology and by increasing levels of intestinal immunoglobulins in weaned piglets (52). His is a conditionally EAA and an important anti-inflammatory factor in the intestinal epithelial cells, and His supplement alleviates colitis of murine (53). Moreover, the decrease of His increases relapsing risk in the emission of ulcerative colitis patients, and it may be a non-invasive predictive marker in intestinal inflammation (54). It must be noted that the levels of Leu and His were significantly lower in the HT group than in the HF group, which might indicate that the intestinal immune defense function in the HT group is lower than that in the CT group. Furthermore, compared with CD, Glu decreased significantly in CT, but its change degree was less than that of HF and HT. Collectively, these results highlight the significant changes of some metabolites, and these changes may exist only in the HF and its colitis model. In addition, changes in serum metabolism between the CT group and the HT group were also observed. EAAs have significant effects on intestinal inflammation (10). It has been reported that Phe manifests beneficial effects in the treatment of IBD by inhibiting *Tnf*α productions and enhancing immune responses (55). In addition, the antioxidant and anti-inflammatory properties of Phe give Phe with chromium a protective effect on indomethacin-induced IBD in rats (56). Phe, as one of the EAAs for humans and animals, was decreased significantly in the HT group compared with the CT group in our study. This change may be caused by colitis in an HF state, which suggests that HF might induce severer colitis compared with CD following TNBS enema.

There exists a highly efficient BA preservation and recycling system within the body, which is termed the enterohepatic circulation (57). BAs, which are biosynthesized by the catabolism of cholesterol in the liver, are involved in maintaining lipid, glucose, and energy metabolism in the liver, intestine, and adipose tissue (58). HF feeding did not disturb BA synthesis regulators, such as FXR-SHP or FXR-FGF15 (data not shown), but one of the key BA synthesis enzymes (*Cyp8b1*) was increased in the liver of HF mice, suggesting that HF may change the ratio of CA and CDCA synthesized by the liver, which leads to disturbed BA metabolism. Through the microscopic MSI analysis of the terminal ileum, we found that HF significantly reduced the distribution of TCA in the ileum tissue section. Interestingly, in the microscopic MSI of colon tissue, we also noticed that HF reduced the distribution of TCA in colon tissue. In view of previous studies have confirmed that TCA has strong anti-inflammatory effects in the gut that control gut bacteria overgrowth and protect intestinal barrier function (26, 27), we speculated that the molecular basis of HF aggravating TNBS-induced colitis is partly to reduce TCA distribution in colonic tissue, so as to reduce the anti-inflammatory ability of colonic mucosal that leads to gut bacteria overgrowth and intestinal barrier dysfunction. The main limitation is that the current study cannot determine which specific changes induced by HF feeding lead to worse IBD in a TNBS model. All the above-mentioned changes in BA, metabolites, or gene expression are to some distance associated with colitis susceptibility. As shown in Figure 5, Phe and C18:2 are strongly associated with AST and ALT, respectively, which indicated the potential role as indicators of colitis formation. Further study will be determined to distinguish the contribution of metabolic changes caused by change of diet to colitis susceptibility.

## Conclusion

In summary, our results clearly indicate the possibility of the adverse effects of short-term HF on the metabolism of mice, including metabolic changes in FAs, AAs, and BAs, which might continue to have negative effects on health and promote the occurrence and development of IBD. Therefore, dietary fat intake is a factor that must be carefully considered, especially in the IBD population. Our results also suggest that HF-primed mice might be more likely to develop abnormal liver function or even hepatitis after TNBS-induced colitis, which needs to be further studied.

## Data Availability Statement

The original contributions presented in the study are included in the article/Supplementary Material, further inquiries can be directed to the corresponding authors.

## Ethics Statement

The animal study was reviewed and approved by the Institutional Animal Care and Use Committee (IACUC) at the China Medical University.

## Author Contributions

H-XL and JD conceived of the study and participated in its design and coordination. Z-HW, JY, LC, and QZ carried out the experimental work and were responsible for analyzing the data. Z-HW, QZ, S-SZ, JY, LC, and X-YW were responsible for animal experiments. Z-HW, YL, and DC involved in manuscript revision. All authors have given approval to the final version of the manuscript.

## Conflict of Interest

The authors declare that the research was conducted in the absence of any commercial or financial relationships that could be construed as a potential conflict of interest.

## Publisher’s Note

All claims expressed in this article are solely those of the authors and do not necessarily represent those of their affiliated organizations, or those of the publisher, the editors and the reviewers. Any product that may be evaluated in this article, or claim that may be made by its manufacturer, is not guaranteed or endorsed by the publisher.

## Supplementary Material

Supplementary Figure 1 **(A)** The mass spectrum and secondary mass spectrometry of TCA standard. **(B)** Secondary mass spectrometry of ileum (precursor ion *m*/*z* 514.284). **(C)** Secondary mass spectrometry of colon (precursor ion *m*/*z* 514.284). Matrix: 9AA.

Supplementary Figure 2 Phenotypes of mice fed a CD and HF for 4 weeks. **(A)** Schematic diagram for colitis model in CD and HF feeding mice. Male C57BL/6 mice were fed with a HF for 4 weeks, and control diet feeding mice were used as normal control. Three weeks later, mice of model group were presensitized with 1% (wt/vol) TNBS solution. Eight days after that, mice were treated under anesthesia with 2.5% (wt/vol) TNBS solution via intrarectal injection for 3 days to induce colitis. pre, before treatment. i.r., intrarectal delivery. The control group fed with CD or HF was not treated with TNBS. **(B)** All mice were sacrificed on day 3, and colons were collected to estimate mucosal damage by detecting colon lengths; gross morphology of the colons on day 3. **(C)** The representative histological sections were observed under microscopy (magnification: 2.5×). **(D)** qPCR quantitation of pro-inflammatory cytokines and chemokines in colonic mucosa on day 3. **(E)** The average daily food intake per mouse during the period of CD or HF feeding. The food intake of mice was recorded twice a week. *n* ≥ 3. The data were shown as mean ± SEM. **p* < 0.05. CD, control diet group; HF, high-fat diet group.

## Abbreviations

BA: bile acid
BAs: bile acids
TNBS: 2,4,6-trinitrobenzenesulfonic acid
CD: control diet
CT: control diet and TNBS administration
HF: high-fat diet
HT: high-fat diet and TNBS administration
MSI: mass spectrometry imaging
NPA: N-1-naphthylphthalic acid
9AA: 9-aminoacridine
DCA: deoxycholic acid
CDCA: chenodeoxycholic acid
UDCA: ursodeoxycholic acid
TDCA: taurodeoxycholic acid
TCDCA: taurochenodeoxycholic acid
TUDCA: tauroursodeoxycholic acid
LCA: lithocholic acid
TLCA: taurolithocholic acid
TCA: taurocholic acid
TMCA: tauromuricholic acid
GCA: glycocholic acid
H&E: hematoxylin and eosin
AAs: amino acids
FAs: fatty acids
IBD: inflammatory bowel disease
MALDI: matrix-assisted laser desorption/ionization
EPA: eicosapentaenoic acid
EAAs: essential amino acids.
