# Supplementary figures and images for: Short-Term High-Fat Diet Fuels Colitis Progression in Mice Associated With Changes in Blood Metabolome and Intestinal Gene Expression
Source: Front Nutr. 2022 Jun 7;9:899829. doi: 10.3389/fnut.2022.899829 (PMC9209758; doi:10.3389/fnut.2022.899829)

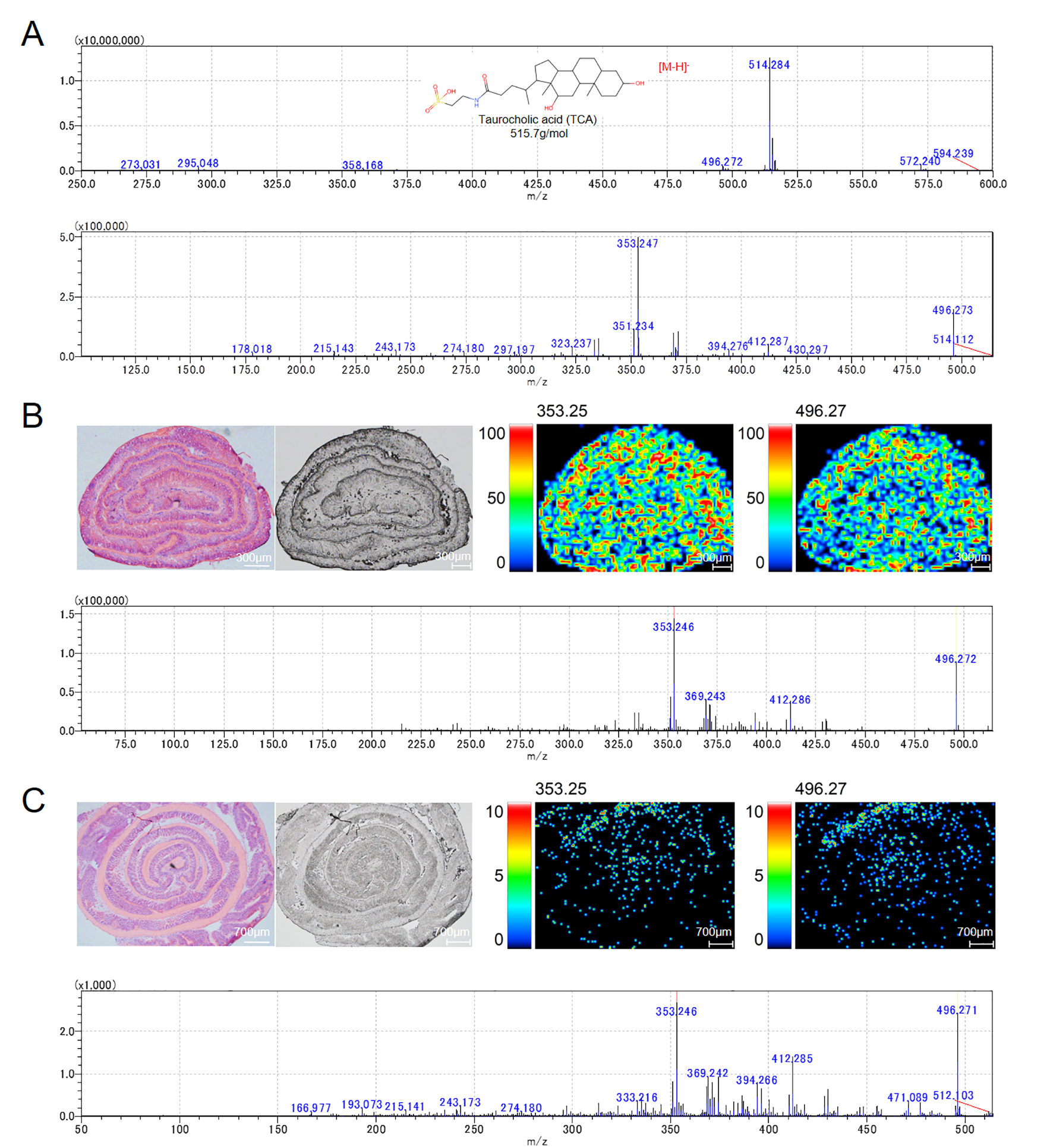

Supplement: Supplementary Figure 1 — (A) The mass spectrum and secondary mass spectrometry of TCA standard. (B) Secondary mass spectrometry of ileum (precursor ion m/z 514.284). (C) Secondary mass spectrometry of colon (precursor ion m/z 514.284). Matrix: 9AA. [file Image_1.tiff]

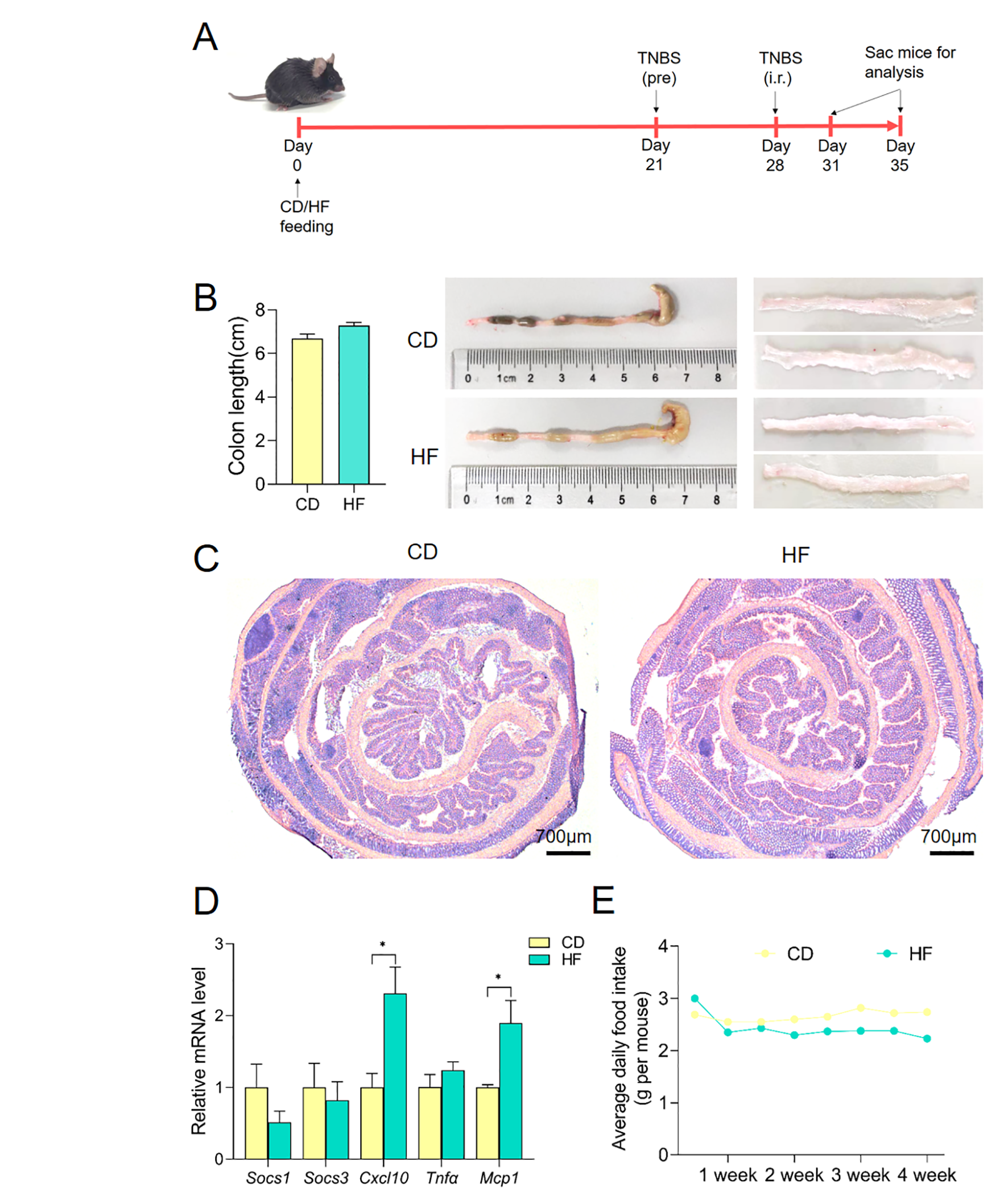

Supplement: Supplementary Figure 2 — Phenotypes of mice fed a CD and HF for 4 weeks. (A) Schematic diagram for colitis model in CD and HF feeding mice. Male C57BL/6 mice were fed with a HF for 4 weeks, and control diet feeding mice were used as normal control. Three weeks later, mice of model group were presensitized with 1% (wt/vol) TNBS solution. Eight days after that, mice were treated under anesthesia with 2.5% (wt/vol) TNBS solution via intrarectal injection for 3 days to induce colitis. pre, before treatment. i.r., intrarectal delivery. The control group fed with CD or HF was not treated with TNBS. (B) All mice were sacrificed on day 3, and colons were collected to estimate mucosal damage by detecting colon lengths; gross morphology of the colons on day 3. (C) The representative histological sections were observed under microscopy (magnification: 2.5×). (D) qPCR quantitation of pro-inflammatory cytokines and chemokines in colonic mucosa on day 3. (E) The average daily food intake per mouse during the period of CD or HF feeding. The food intake of mice was recorded twice a week. n ≥ 3. The data were shown as mean ± SEM. *p < 0.05. CD, control diet group; HF, high-fat diet group. [file Image_2.tiff]
