# Supplementary material for: Short-Term High-Fat Diet Fuels Colitis Progression in Mice Associated With Changes in Blood Metabolome and Intestinal Gene Expression
Source: Front Nutr. 2022 Jun 7;9:899829. doi: 10.3389/fnut.2022.899829 (PMC9209758; doi:10.3389/fnut.2022.899829)
Supplement: Supplementary file 3 [file Table_1.docx]

**Supplementary table 1:** The nucleotide sequences of primers for q-PCR

| Primers | Forward (5’-3’) | Reverse (5’-3’) |
| --- | --- | --- |
| *Acly* | CAGGAACTGTGGCTCCTTCAC | AGGGCTCCAATGTCAACATATTC |
| *Acc1* | ATGTCCGCACTGACTGTAACCA | TGCTCCGCACAGATTCTTCA |
| *Fasn* | CCCGGAGTCGCTTGAGTATATT | GGACCGAGTAATGCCATTCAG |
| *Scd1* | CGTTCCAGAATGACGTGTACGA | AGGGTCGGCGTGTGTTTC |
| *Pparα* | CTGCAGAGCAACCATCCAGAT | GCCGAAGGTCCACCATTTT |
| *Cpt1a* | GGAGACTTCCAACGCATGACA | AATTTGTGGCCCACCAGGAT |
| *Cpt2* | CAACTCGTATACCCAAACCCAGTC | GTTCCCATCTTGATCGAGGACATC |
| *Cd36* | AGGTCTATCTACGCTGTGTTCGGA | CAATGGTTGTCTGGATTCTGGAGGG |
| *Acat1* | GCAGGCTTACCTATTTCTACTC | CAGTTAGCCCGTCTTTTACAATC |
| *Pepck* | TGTCGGAAGAGGACTTTGAGAAA | TGCTGAATGGGATGACATACATG |
| *Srebp1c* | TGTGATCTACTTCTTGTGGCCCGT | AGGCTGCTCAGGTCATGTTGGAAA |
| *Tgr5* | CTGCCCAAAGGTGTCTACGA | GCATTGGCTACTGGTGTGGT |
| *Cyp7a1* | GAGCCCTCAAGCAATGAAAG | GCTGTCCGGATATTCAAGGA |
| *Cyp7b1* | TTGTAGCCCTCTTTCCTCCA | CTTGTTCCGAGTCCAAAAGG |
| *Cyp8b1* | GGACAGCCTATCCTTGGTGA | GACGGAACTTCCTGAACAGC |
| *Cyp27a1* | GAGAGTGAATCAGGGGACCA | CCATTTGGGAAGGAAAGTGA |
| *Gapdh*  *Socs1*  *Socs3*  *Cxcl10*  *Tnfα*  *Mcp1* | TGTGTCCGTCGTGGATCTGA  GACACTCACTTCCGCACCTT  GCCACCTGGACTCCTATGAGAA  TCTCTCCATCACTCCCCTTTACC  TCAGCCTCTTCTCATTCCTG  CAAGAAGGAATGGGTCCAGA | CCTGCTTCACCACCTTCTTGA  CGAAGAAGCAGTTCCGTTG  GAGCATCATACTGATCCAGGAACTC  CTTGCTTCGGCAGTTACTTTTGTC  CAGGCTTGTCACTCGAATTT  TGAGGTGGTTGTGGAAAAGG |
